# Development of an In Vitro Propagation Protocol and a Sequence Characterized Amplified Region (SCAR) Marker of *Viola serpens* Wall. ex Ging

**DOI:** 10.3390/plants9020246

**Published:** 2020-02-14

**Authors:** Shipra Rani Jha, Ruphi Naz, Ambreen Asif, Mohammad K. Okla, Walid Soufan, Abdullah A. Al-Ghamdi, Altaf Ahmad

**Affiliations:** 1 Department of Botany, Jamia Hamdard, New Delhi 110062, India; ahmadaltaf@rediffmail.com; 2 Department of Botany, Aligarh Muslim University, Aligarh 202002, India; rnaz104@myamu.ac.in (R.N.); aasif.rs@amu.ac.in (A.A.); 3 Botany and Microbiology Department, College of Science, King Saud University, P.O. Box. 2460, Riyadh 11451, Saudi Arabia; malokla@ksu.edu.sa (M.K.O.); abdaalghamdi@ksu.edu.sa (A.A.A.-G.); 4 Plant Production Department, Faculty of Food and Agricultural Sciences, King Saud University, P.O. Box 2460, Riyadh 11451, Saudi Arabia; waoufan@ksu.edu.sa

**Keywords:** micropropagation, conservation, secondary metabolites, acclimatization, SCAR marker, *Viola serpens*

## Abstract

An efficient protocol of plant regeneration through indirect organogenesis in *Viola serpens* was developed in the present study. Culture of leaf explants on MS (Murashige and Skoog) medium supplemented with 2.0 mg/L 6-benzyladenine and 0.13 mg/L 2,4-dichloro phenoxy acetic acid. Adventitious shoot formation was observed when calli were transferred on to MS medium containing 0.5 mg/L α-naphthalene acetic acid and 2.25 mg/L kinetin, which showed the maximum 86% shoot regeneration frequency. The highest root frequency (80.92%) with the 5.6 roots per explant and 1.87 cm root length was observed on MS medium supplemented with 2 mg/L indole-3-butyric acid. The plantlets were transferred to the mixture of sand, coffee husk and soil in the ratio of 1:2:1 in a pot, and placed under 80% shade net for one month. It was then transferred to 30% shade net for another one month, prior to transplantation in the field. These plantlets successfully acclimatized under field conditions. A Sequence Characterized Amplified Region (SCAR) marker was also developed using a 1135 bp amplicon that was obtained from RAPD (Random Amplification of Polymorphic DNA) analysis of six accessions of *V. serpens.* Testing of several market samples of *V. serpens* using the SCAR marker revealed successful identification of the genuine samples of *V. serpens.* This study, therefore, provides a proficient in vitro propagation protocol of *V. serpens* using leaf explants and a SCAR marker for the authentic identification of *V. serpens*. This study will be helpful for conservation of authentic *V. serpens*.

## 1. Introduction

Medicinal plants have long been used as an important source of life saving drugs for human beings. India is a rich source of medicinal plants, but rapid population growth and human activities are now destroying their natural environment. Various medicinal plants are facing extinction and they require some urgent steps for their conservation. *Viola serpens* (Violaceae) is an important medicinal plant [1]. It has a high medicinal value in skin diseases, bleeding piles, throat cancer, asthma, constipation, fever, headache, and cough [2,3]. In addition, *V. serpens* is the main ingredient of Joshanda, used in cough and cold [4]. India, China, Java, Ceylon, Philippines, and Thailand are the geographical distribution of *V. serpens* in the world. In India, it is distributed in the Himalayan region, the hills of Meghalaya, Nagaland, and Manipur [5]. The hilly regions of Orissa, Himachal Pradesh, Uttarakhand, Karnataka and Tamil Nadu are other sources of *V. serpens* in India [6]. *Viola serpens* is a perennial herb, occurring in both cluster or solitary forms. Leaves that are ovate-lance shaped originate directly from the creeping roots as the stem is very short. Flowers are lilac in color, pods are pale brown, and seeds are tiny and black. Phytochemical investigations of *V. serpens* revealed the presence of tannins, flavonoids, reducing sugars, terpenoids, amino acids [7], methyl salicylate, saponin, mucilage, glycoside, voiline, and quercitrin [8]. The plant is commonly propagated through seeds and cuttings. As the cool climate is suitable for its healthy growth and blooming, it is grown at high altitude [9]. Since the commercial demand for this plant is high in pharmaceutical industries, overwhelming exploitation of *V. serpens* from wild resources has adversely affected its natural regeneration. As a consequence, the population of this plant is decreasing at alarming rate. Therefore, there is an urgent need to establish a stable and cost effective propagation system for the germplasm conservation of this species.

To overcome the present situation, in vitro propagation holds tremendous potential for the production of good quality plant-based medicines and is also an important tool for the conservation of the critical genotypes of the plants. This technology attained recognition in plant biotechnology for the fruitful micropropagation of plant species and their improvement for commercial application. Tissue culture techniques provide a safe way for exchanging the plant material globally, require minimum space to establish the most plants, and promote molecular investigations and ecological studies [10]. In addition, secondary metabolites produced in the plants are not only beneficial to the plant itself, but also are exploited because of their medicinal values. Secondary metabolites are generally produced in lesser amounts in plants. Therefore, various in vitro cell and organ cultures have been performed to increase their production. This technique has received considerable attention from plant biotechnologists to produce vital bioactive compounds and phytochemicals in eco-friendly conditions.

Increased demand for medicinal plants for pharmaceuticals, cosmetics, and other products are also causing adulteration or substitution to herbal medicines. Adulteration or substitution of a herbal drug may be intentional for monetary gain or unintentional due to lack of knowledge and awareness about medicinal plants and their properties. Use of the wrong herb or adulterated plant material can be harmful or it may worsen the condition in the treatment of various ailments and, sometimes, it may cause death. These adulterants do not contain the secondary metabolites present in the genuine medicinal plant. Authentication and identification of the genuine medicinal plant has become a critical issue as adulteration of herbal drugs is increasing daily. Therefore, the correct identification, authentication, and quality assurance of the genuine plant have become essential requirements to maintain the quality, safety, and efficacy of the medicinal plant or drug [11]. Molecular biology offers various techniques that can be useful for the correct identification and authentication of a plant. Since the genetic composition of all living organism is unique, development of a marker, based on genomic DNA composition, can be more reliable for the authentic identification of medicinal plants. Such markers are advantageous over phenotypic markers because genomic DNA composition is not dependent on environmental and physiological conditions [12]. The efficiency of DNA-based molecular markers is dependent on the genetic polymorphism detected in the given accessions of the plants [13].

In the present study, an efficient and reproducible propagation protocol for *V. serpens* was established using leaf explants and a DNA-based reliable and reproducible marker was developed for the correct identification of genuine *V. serpens*.

## 2. Results

### 2.1. Callus Induction

**Figure 1:**
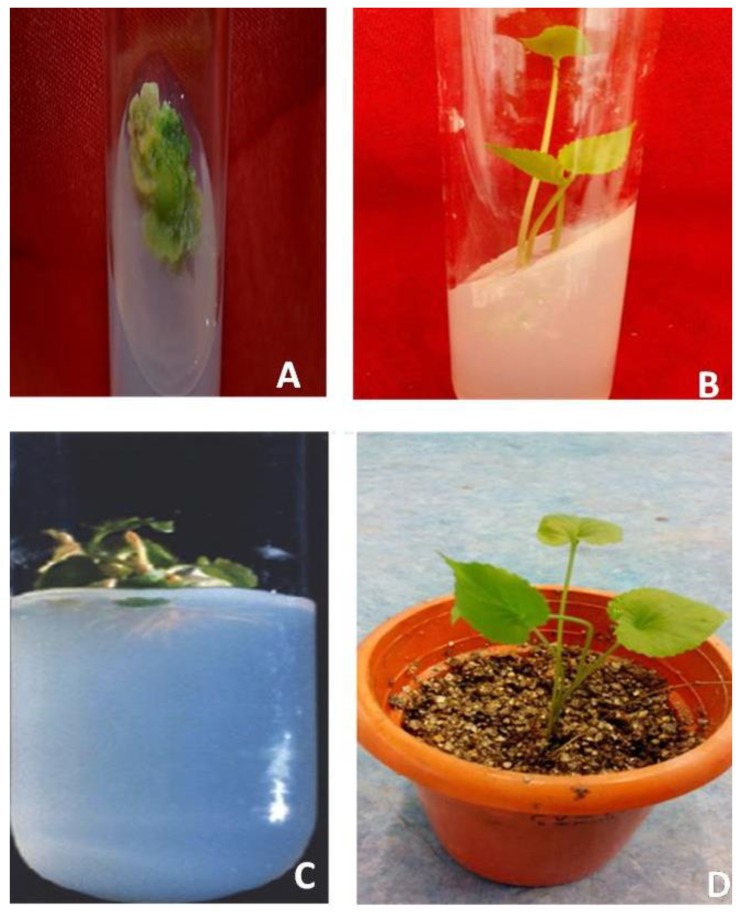
(**A**) Callus induction (*Viola serpens*) on MS medium supplemented with BAP (2.0 mg/L) + 2, 4- D (0.13 mg/L); (**B**) induction of the shoot on MS medium augmented with NAA (0.50 mg/L) + KIN (2.25 mg/L); (**C**) root induction on MS medium with IBA (2.0 mg/L); and (**D**) hardening of plantlets.

Sterilized leaf explants were inoculated on MS medium. Varying concentrations of plant growth regulators (2,4-dichloro phenoxy acetic acid, 2,4-D; α-naphthalene acetic acid, NAA; 6-benzyladenine, BAP; indole-3-acetic acid, IAA; indole-3-butyric acid, IBA; and kinetin, KIN) were supplemented with the MS medium (Supplementary Table S1). After inoculation of the explant, observations were taken from day 25 up to day 60. No callusing of the explants was reported when the plant growth reguators (PGRs) were supplied without any combination (Table 1). Only a 10% frequency of callus formation appeared on the MS medium augmented with 2.0 mg/L BAP (Supplementary Table S1). Different combinations of BAP (2.0 mg/L BAP) with various concentrations of 2,4-D, BAP (2.0 mg/L) with IAA (0.25–1.0 mg/L,) or BAP (2.0 mg/L) in combination with 2,4-D (1.0 mg/L) and KIN (0.1–0.5 mg/L) were tested for callus formation (Table 1). After 5–8 weeks of inoculation, initiation of callus appeared on MS medium along with 1.0–2.5 mg/L BAP and 0.10–0.15 mg/L 2,4-D or BAP (1.0–1.5 mg/L) in combination with 2,4-D (1.0 mg/L) and KIN (0.1–0.5 mg/L) (Table 1). Subsequent subculture in the same medium (MS + BAP + 2,4-D or MS + BAP + 2,4-D + KIN) resulted in increased growth of the callus. The callus appeared green (Figure 1A). Compared to both combinations, the best results for callusing were detected on the MS medium supplemented with 2.0 mg/L BAP in combination with 0.13 mg/L 2,4-D. Eighty-eight percent callus formation from the leaf explants was observed with this combination after 45 days of inoculation, whereas only about 45% callus was obtained on the MS + BAP + 2,4-D + KIN after 60 days of inoculation (Table 1).

### 2.2. Induction of Multiple Shoots

Subculturing of the callus, obtained from MS + BAP + 2,4-D media, was carried out on MS medium containing auxin and cytokinin in different combinations (Table 2). Initiation of shoot buds were observed on the MS medium containing 0.5 mg/L NAA and 1.5–2.5 mg/L KIN. The MS medium containing 0.5 mg/L NAA and 2.25 mg/L KIN gave the optimum 70% shoot regeneration frequency. Subculturing of the shoot clumps on this medium resulted in an incerased formation of the shoot. Fifth subculture passages on this culture medium resulted in the highest regeneration frequency (86%) of shoots and the highest number of shoots (12), which was higher than the combination of NAA (0.50 mg/L) along with BAP (1.0–2.0 mg/L) (Table 2, Figure 1B). Although, different concentration of MS + NAA (1.0, 1.5 and 2.0 mg/L) and KIN (3.0, 3.5 and 4.0 mg/L) were tried, no further enhancement of shoot regeneration frequency was observed (Data not shown). Thus, the combination of MS + NAA (0.5 mg/L) and KIN (2.25 mg/L) was considered to be the optimal growth regulator combination for maximum shoot regeneration in *V. serpens* (Table 2).

### 2.3. In Vitro Rooting as Influenced by Plant Growth Regulators

In vitro grown shoots (5.1 cm) were cu, and placed on the MS medium alone without any PGR or with different concentrations of PGRs (IAA and IBA). Root induction was not observed when the shoot was transferred on the MS (Full or 1/2) medium alone. The highest frequency of rooting was observed after 2–3 weeks of inoculation on the MS medium containing various concentrations of IBA. Among the different concentrations of IBA, the highest percentage of rooting response (81%) along with the mean number of roots (5.6) and root length (1.87 cm) was found on the MS medium containing 2 mg/L IBA (Table 3, Figure 1C). Shoots transferred on the MS medium containing various concentrations of IAA exhibited a lesser number of root formations than IBA (Table 3).

### 2.4. Acclimatization

Elongated shoots with well-defined roots were used for acclimatization. Individual plantlets were taken from the flasks. These plantlets, after washing with tap water, were shifted to a mixture of sand, decomposed coffee husk, and soil in a ratio of 1:2:1 in sterilized pots. They were kept for 15 days under high humidity, i.e., 70–80%, to avoid desiccation. Thereafter, the plantlets were placed under 80% shade net for one month. Therafter, these plants were transferred to the field (Figure 1D).

### 2.5. Sequence Characterized Amplified Region (SCAR) Development

**Figure 2:**
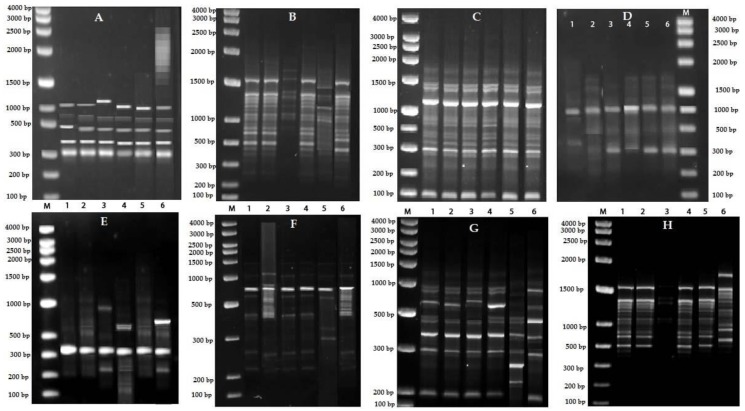
RAPD profile of *V. serpens* with (**A**) OPAA-01, (**B**) OPAA-03, (**C**) OPAA-04, (**D**) OPAA-07, (**E**) OPAA-08, (**F**) OPAA-09, (**G**) OPAA-10, and (**H**) BG-02 primers. Lane M = DNA ladder. Lanes 1–6 are accessions of *V. serpens.*

Accessions of *V. serpens* were procured from different sources for RAPD (Randomly Amplified Polymorphic DNA) analysis (Table 4). Out of 20 RAPD primers (Supplementary Table S2), amplification of the genomic DNA of all the 6 accessions of *V. serpens* was observed with only 8 primers, producing distinct, good quality, reproducible fingerprint patterns (Figure 2, Supplementary Table S3).

**Figure 3:**
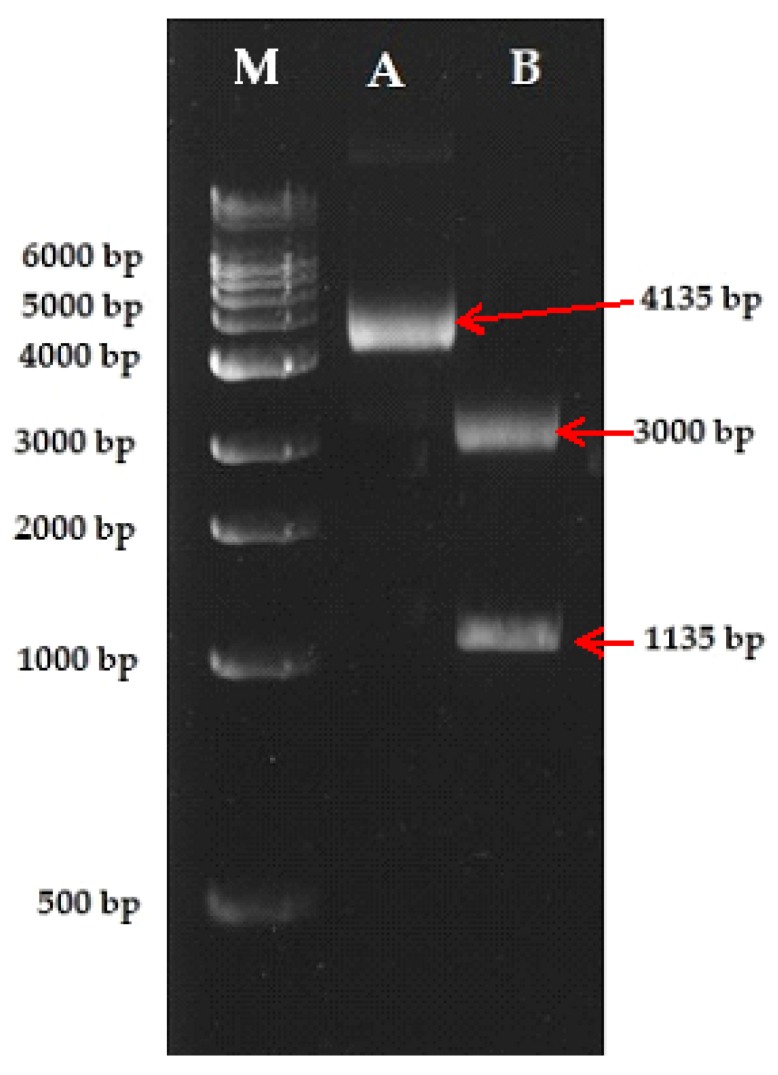
Confirmation of the cloning of the RAPD amplicon in pGEMT-easy vector by restriction digestion. Lane A = cloned RAPD amplicon in pGEMT-easy vector (undigested), Lane B = digestion with *Eco*RI, generating fragments of pGEMT-easy vector (3000 bp) and RAPD amplicon (1135 bp).

A bright and distinct RAPD amplicon (~1135 bp) that was specific to all the six accessions of *V. serpens*, as obtained with the OPAA-04 RAPD primer (Figure 2C), was eluted and cloned in the pGEM-T easy vector (Promega, Madison, WI, USA). The size of the recombinant DNA (vector + RAPD amplicon) was ~4335 bp. The recombinant DNA successfully transformed in *Escherichia coli*. The isolated recombinant DNA was resolved on 1.0% agarose gel. The size of the isolated plasmid was ~4335 bp. Restriction digestion analysis with *Eco*R1 revealed a band of ~1135 and 3000 bp, confirming the cloning of the RAPD amplicon (Figure 3).

**Figure 4:**
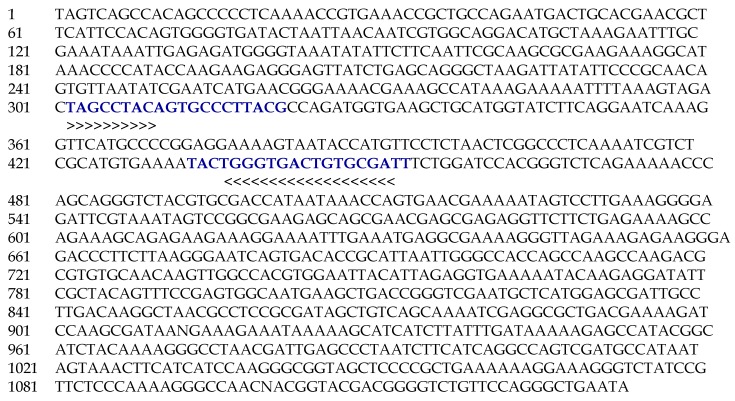
Sequence of cloned RAPD amplicon. Blue colored nucleotides show primer positions.

**Figure 5:**
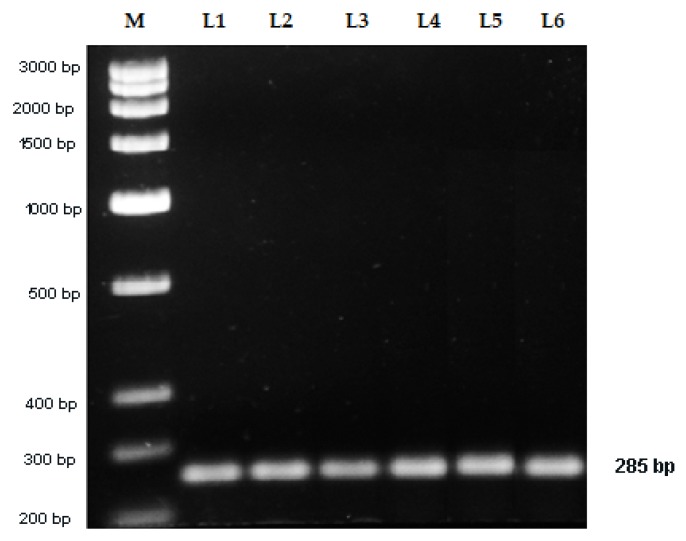
Amplification of accessions of *Viola serpens* using SCAR primers (Vio F and Vio R). Lane M = DNA marker, Lane L1–L6 = Samples of *Viola serpens.*

Sanger sequencing of the recombinant DNA was done using SP6 and T7 primers (Figure 4). Forward (Vio F) and reverse (Vio R) SCAR primers were designed taking the sequence of the RAPD amplicon (Table 5). The expected fragment size of the amplified DNA with these primers was 285 bp. When the genomic DNA of all the accessions of *V. serpens* was amplified with these primers, a single and bright 285 bp fragment of DNA was obtained (Figure 5). It confirmed that the designed SCAR primers are highly specific and sensitive towards *V. serpens*.

**Figure 6:**
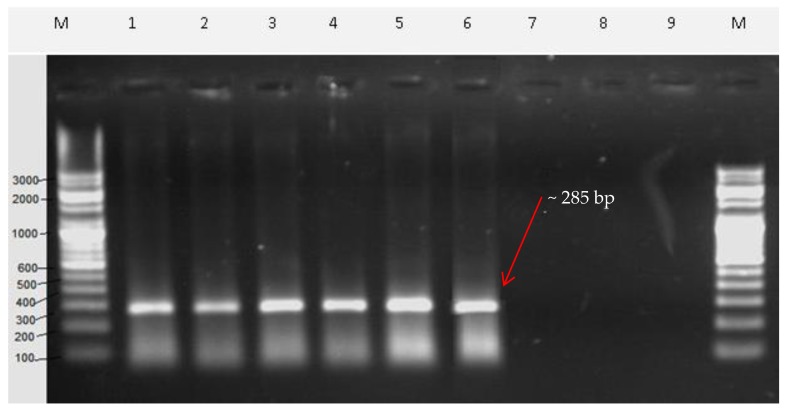
Identification of market samples of *Viola serpens* with SCAR primers (Vio F & Vio R). Lane M = 100 bp standard DNA marker, Lanes 1–9 = Market samples of *Viola serpens.*

To test the applicability of the SCAR marker for the authentication of *V. serpens*, the SCAR primers were used to amplify genomic DNA of commercial crude drug market samples. The market samples in the lane numbers 1–6 of agarose gel showed a single and distinct DNA band of 285 bp (Figure 6). No amplified product was seen in the lane numbers 7–9 of the agarose gel (Figure 6). Therefore, it was found that the developed SCAR primers were able to amplify the genomic DNA of *Viola serpens* from the commercially available crude drug (Lanes 1–6). Absence of the amplified product in the rest of the lanes showed that the commercial crude drug did not contain *Viola serpens.* These results show the commercial applicability of the SCAR primers and, thus, it can be used for the molecular authentication of medicinal plants.

## 3. Discussion

Increases in the population and rapid technological advances are putting tremendous pressure on natural genetic resources causing wild resources of medicinal plants to diminish rapidly. Micropropagation is a key tool of plant biotechnology that exploits the totipotent nature of plant cells, a concept proposed by Haberlandt [14]. In vitro micropropagation of valuable plant species plays a major role in the conservation of the germplasm, rapid clonal propagation of genetically-manipulated elite clones, production of secondary metabolites, establishment of extensive collections using minimum space, and the supply of valuable material for wild population recovery [10,15]. This technique is widely used for the commercial propagation of various medicinal plants [16,17]. In the present study, in vitro micropropagation protocol of *V. serpens* was established using leaf explants because this plant is widely used in a large number of herbal formulations and the procurement of this plant is carried out mainly from wild resources. Earlier, plant propagation protocols for *V. patrinii* [18,19] and *V. ulginosa* [20] were established. In vitro propagation of *V. serpens* was also carried out earlier using petiole explants [4]. In our study, leaf explants were inoculated on MS medium without and with varying concentrations of PGRs (NAA, KIN, BAP, 2,4-D, IAA) singly. No positive response in the callusing of the explants was found. The addition of auxin along with cytokinin produced positive results in *V. serpens*. Among the different combination tested, supplementation with a combination of BAP (2.0 mg/L) and 2,4-D (0.13 mg/L) with the MS medium resulted in the best callus induction, showing about 90% callus formation from the leaf explants. Further, the MS medium augmented with 0.5 mg/L NAA and 2.25 mg/L KIN gave the best results in our study for shoot proliferation from the callus. In contrast with our results, Soni and Kaur [21] showed that MS medium in combination with BA (1.0 mg/L) and KIN (0.25 mg/L) exhibited the best response for in vitro shoot regeneration in *V. pilosa.* Vishwakarma et al. [4] reported that the petiole explants did not show any sign of callus formation on MS medium supplemented with different PGRs (NAA, BAP, KIN, IAA) singly in *V. serpens*. However, the addition of auxin along with cytokinin gave positive results in petiole explant. The MS medium supplemented with 2,4-D produced a significant result using unfertilized ovules in *Viola odorata* [22]. Chalgeri et al. [18] reported no response on callus induction of *V. patrinii* with higher concentrations of NAA and BAP. In addition, transfer of regenerated shoot on half-strength MS medium augmented with IBA induced root formation, where 73% rooting with a 3.6 average number of roots and 1.3 cm root length was observed. These results are better than earlier reports on root induction in *V. serpens* using IAA [4]. In *Viola patrinii*, development of significant roots were obtained in 2-fold diluted MS media supplemented with 9.85 μM IBA and 2% (*w*/*v*) sucrose [18]. The highest rooting frequency was obtained on MS medium supplemented with 2.0 mg/L IBA in our study after 2–3 weeks of transfer. Complete plantlets were successfully acclimatized in the culture room and then transferred in field conditions. The in vitro-micropropagated plants successfully survived the natural environmental conditions in the field, indicating the success of this protocol for plant regeneration of *V. serpens* using leaf explants.

In addition to the development of an efficient in vitro propagation protocol for *V. serpens,* a DNA-based technique for the authentication of genuine *V. serpens* was also developed in this study in view of the need for a tool that can distinguish a genuine plant from the adulterated samples. The differences that distinguish one species from another are the deoxyribonucleic acids (DNA), which are encoded in the genetic material of the species and these DNA-based methods are one of the most reliable methods for the authentication of plants. Thus, these DNA-based markers are unique and stable as the DNA of every individual is unique in nature. Many molecular markers, viz., RAPD, RFLP (Restriction Fragment Length Polymorphism), and AFLP (Amplified Fragment Length Polymorphism), have been used for the DNA analysis of medicinal plants. These molecular markers are easy to develop and are simple markers but the lack of reproducibility makes them less reliable for the identification of the genuine plant. To overcome these drawbacks, a more specific marker (i.e., SCAR marker) was developed as it was single locus-specific and its PCR amplification was not very sensitive to the reaction conditions. Conversion of molecular markers, like RAPD and RFLP, to the sequenced data-based SCAR marker increases the reproducibility of PCR products. Thus, this marker is a fast and more reliable method for the authentication of the plant. In the present study, a SCAR marker was developed for the identification of *V. serpens.* SCAR markers have also been used to study adulteration in medicinal plants like *Cynanchum* [23], *Echinacea* [24], *Pueraria* [25], *Gardenia jasminoides* [26], and *Phyllanthus* species [27]. Authentication of commercially important food and spice products, like ground chili [28], has also been done using SCAR markers. *Viola serpens* is a priority-listed medicinal plant. Because of over exploitation, availability of this plant is continuously decreasing in the wild. Thus, development of a SCAR marker for its identification will help to conserve natural resources. Authentication of endangered plants, like *Michelia coriacea* [29], *Commiphora* spp. [30], and *Physalis* [31], has also been carried out earlier using SCAR markers.

## 4. Materials and Methods

### 4.1. Collection of Plant Materials

For micropropagation, authentic plants of *Viola serpens* were procured from Jogindernagar, Himachal Pradesh, India and grown at Jamia Hamdard herbal garden, New Delhi. For developing the SCAR marker, plants were collected from six different locations (Table 4). Authentication was done by the Botanical Survey of India, Dehradun (India) with a voucher herbarium specimen No. 114835 of *V. serpens*.

### 4.2. Micropropagation of V. serpens

Leaf primordial tissues were collected from the plant and washed under tap water 3–4 times. These explants were soaked in a 5% Teepol solution (a liquid detergent; Qualigens Fine Chemicals, Mumbai, India), washed under running water followed by surface sterilization with mercuric chloride (0.1%) for 1 min, then washed several times with sterile water. The explants were then inoculated on the sterilized MS medium [32] alone or on the MS medium (pH 5.7) containing various concentrations of PGRs, like 2,4-dichloro phenoxy acetic acid (2,4-D), α-naphthalene acetic acid (NAA), 6-benzyladenine (BAP), indole-3-aetic acid (IAA), indole-3-butyric acid (IBA), and kinetin (KIN), for callus induction. The callus obtained was subcultured on the MS medium supplemented with different concentrations of NAA, KIN, BAP alone, and a combination of NAA + BAP or NAA + KIN for shoot differentiation. Emerging shoots from explants were inoculated on half- or full-strength MS medium supplemented with varying concentrations of IAA and IBA (0.5, 1.0, 1.5, 2.0, and 2.5 mg/L) for rooting. Maintenance of the cultures was carried out at 25 °C ± 2 °C with an 8/16 h (light/dark) photoperiod.

Individual plantlets of *V. serpens* were excised from the flasks, then washed with tap water, and transferred to a mixture of sand, decomposed coffee husk, and soil in a ratio of 1:2:1 in a sterilized pot. High humidity (80–90%) conditions were kept for 15 days to prevent desiccation. The experiments were carried out, taking six replicates, with each plant growth regulator. Data from the experiment were analyzed by calculating the mean and standard error using SYSTAT 13 software (Stat Soft Inc., Tulsa, OK, USA).

### 4.3. Development of Sequence Characterized Amplified Region (SCAR)

Genomic DNA isolation of *Viola serpens* was carried out by Doyle and Doyle [33]. Twenty random primers (15 from Operon technologies, Qiagen, Germantown, MD, USA and five primers from BangloreGenei, Bengaluru, India) were initially screened for RAPD analysis (Supplementary Table S2). Based on the ability to detect distinct polymorphic amplified products across the accessions, primers were selected for further analysis. The primers that generated weak products were discarded to ensure reproducibility. The RAPD reaction was performed by the method developed by McClelland et al. [34]. The polymerase chain reactions consisted of reaction buffer (20 mM Tris-HCl (pH 8.4), 50 mM KCl, 0.5 U of *Taq* polymerase), 300 mM dNTPs (deoxyribonucleotides), 1.5 mM MgCl_2_, 25 pM primer, and 25 ng template DNA. Amplification conditions were set as an initial denaturation temperature of 95 °C for 4 min, followed by 40 cycles of 94 °C (30 s), 34 °C (50 s), and 72 °C (60 s) in a thermal cycler (Master cycler, Eppendorf, USA). Amplified products were run on 1.0% agarose gel in 1×TAE buffer. A standard 100 bp DNA ladder was also run along with the amplified products. Staining was done by 0.5 μg/mL ethidium bromide. Visualization and photography of the resolved DNA were carried out using a gel documentation system (Alpha innotech, San Leandro, CA, USA). A 1135 bp-sized DNA fragment of *V. serpens* was excised from the agarose gel and eluted using a gel extraction kit (Qiagen, Germantown, MD, USA). Cloning and sequencing of the eluted amplicon were further performed. A-tailing to the amplicon was carried out using the eluted DNA fragment, reaction buffer (1.5 mM MgCl_2_, 0.2 mM dATP), and 1 unit of *Taq* polymerase. The reaction was incubated for 30 min at 70 °C. These A-tailed DNA were ligated into the pGEM-T easy vector (Promega, Madison, WI, USA) following the manufacturer’s instruction. The recombinant DNA was transformed into *E. coli* DH10β. Few distinct colonies were picked and grown on Lauri–Bertani medium containing ampicillin until the OD_600_ reached 0.3. The recombinant DNA was isolated using a plasmid isolation kit (Qiagen, Hilden, Germany). Sequencing of the cloned DNA fragment was performed with SP6 and T7 primers at the Centre for Genomic Application, New Delhi, India. Forward primer (Vio F) and reverse primer (Vio R) for developing the SCAR marker were designed (Table 5). GC contents, secondary structures, and the melting temperature of each primer were also determined. These sequences were custom synthesized by IDT Technologies, USA. The in-house designed SCAR primers were used for the amplification of the genomic DNA of genuine plant materials. Nine samples from a local market in New Delhi, India were procured, which were sold under the name Banafsa (local name of *V. serpens*) (Supplementary Table S4). Genomic DNA of these samples was isolated and amplification of the isolated DNA was carried out using SCAR primers (Vio F and Vio R) under similar PCR conditions. The amplified DNA products were run on 1.0% agarose gel. A standard 100 bp DNA ladder was also run along with the amplified DNA products. Staining was carried out using 0.5 μg/mL ethidium bromide. Visualization and photography of the resolved DNA were carried out using a gel documentation system (Alpha innotech, San Leandro, CA, USA).

## 5. Conclusions

The current investigation provided a promising technique for an efficient regeneration protocol of *V. serpens* using leaf explants. The PGR-free MS medium and the MS medium combined with varying concentrations of single plant growth regulators (NAA, BAP, KIN, IAA, 2,4-D) showed negligible callusing on the explants. The MS medium along with BAP (2.0 mg/L) in combination with 2,4-D (0.13 mg/L) was the best for callusing, where about 90% callus were induced from the leaf explants. Further, the MS medium augmented with NAA (0.5 mg/L) and KIN (2.25 mg/L) gave the best results for shoot proliferation from the callus. Regenerated shoots were rooted on various concentration of IBA. Among the different concentrations of IBA tested, the highest rooting frequency was obtained on MS medium supplemented with IBA (2.0 mg/L) after 2–3 weeks of transfer. Complete plantlets were successfully acclimatized in the culture room and then transferred to the field. Further, a reliable and reproducible marker (SCAR marker) was developed for the identification of genuine samples of the *V. serpens*. This marker can specifically identify the genetic materials of *V. serpens* from other plant species. Therefore, the development of an efficient regeneration protocol and the SCAR marker of *V. serpens* will greatly contribute to the ecological conservation of this important medicinal plant.

## Figures and Tables

**Table 1:** Effect of plant growth regulators (PGRs) on leaf explants of *Viola serpens* for callus formation.

Plant Growth Regulators	Concentrations (mg/L)	Callusing	No. of Days	Percentage of Explants Responded (%)
**MS Medium**	**-**	-	**50–60 Days**	**0**
MS + NAA	0.25 0.50 0.75 0.85 1.00	- - - - -	50–60 days	0
MS + BAP	0.10 0.25 0.50 0.75 1.00	- - - - -	50–60 days	0
MS + 2,4-D	0.10 0.11 0.25 0.35 0.40 0.50	- - - + -	25 days	10
MS + KIN	0.10 0.25 0.50 0.75 1.00	- - - - -	50–60 days	0
MS + BAP + 2,4-D	2.0 + 0.11 2.0 + 0.12 2.0 + 0.13 2.0 + 0.14 2.0 + 0.15	+ - + + +	45 days	20 - 88.5 75 60
MS + BAP + IAA	2.0 + 0.25 2.0 + 0.50 2.0 + 0.50 2.0 + 0.75 2.0 + 1.00	- - - - +	25 days	- - - - 12
MS + BAP + 2,4- D + KIN	2.0 + 1.0 + 0.1 2.0 + 1.0 + 0.2 2.0 + 1.0 + 0.3 2.0 + 1.0 + 0.4 2.0 + 1.0 + 0.5	+ + - - +	50–60 days	40 35 - - 45
MS + NAA + KIN	2.0 + 0.8 2.2 + 0.8 2.3 + 0.9 2.0 + 0.5 2.5 + 0.5	+ + - - -	40 days	45 38 - - -

(+) sign denotes degree of callusing; (-) sign shows no response.

**Table 2:** Shoots regeneration from the calli of *Viola serpens* as influenced by plant growth regulators.

Plant Growth Regulators	Concentrations (mg/L)	Number of Shoots after Fifth Subculture (Mean ± SE)	Shoot Length (cm) (Mean ± SE)	Frequency of Shoots (%)
MS + NAA	0.10	-	-	-
	0.20	-	-	-
	0.25	-	-	-
	0.50	1.03 ± 0.34	1.02 ± 0.05	6
MS + KIN	1.00	-	-	-
	1.50	-	-	-
	1.75	-	-	-
	2.50	-	-	-
MS + BAP	1.00	-	-	-
	1.25	-	-	-
	1.50	2.15 ± 0.48	1.85 ± 0.20	8
	2.00	1.06 ± 0.67	2.07 ± 0.31	6
MS + NAA + BAP	0.50 + 1.00	3.33 ± 0.33	1.05 ± 0.18	10
	0.50 + 1.50	5.60 ± 0.48	3.34 ± 0.27	49
	0.50 + 1.75	3.50 ± 0.22	2.45 ± 0.14	15
	0.50 + 2.00	3.98 ± 0.43	2.67 ± 0.20	17
MS + NAA + KIN	0.50 + 1.50	3.25 ± 0.20	2.25 ± 0.12	12
	0.50 + 1.75	3.80 ± 0.29	2.56 ± 0.11	23
	0.50 + 2.00	4.20 ± 0.32	2.80 ± 0.12	36
	0.50 + 2.25	12.08 ± 0.79	5.96 ± 0.25	86

(-) showed no response. Values are mean of three independent experiments, SE = Standard error.

**Table 3:** In vitro root induction from callus-induced shoots as influenced by IAA or IBA.

Plant Growth Regulators	Concentration	Number of Roots/Explants (Mean ± SE)	Root Length (cm) (Mean ± SE)	Rooting Response (%)
MS plain media	-	-	-	-
IAA	0.5	-	-	-
	1.0	-	-	-
	1.5	-	-	-
	2.0	1.85 ± 0.37	0.71 ± 0.05	34
	2.5	1.50 ± 0.21	1.370 ± 0.10	29
IBA	0.5	-	-	-
	1.0	1.19 ± 0.49	0.51 ± 0.05	64
	1.5	2.80 ± 0.50	1.06 ± 0.08	56
	2.0	5.67 ± 3.70	1.87 ± 0.32	81
	2.5	2.50 ± 0.29	0.65 ± 0.33	65

(-) showed no response. Values are mean of three experiments, SE = Standard error.

**Table 4:** Sources of *Viola serpens.*

Plant Name	Collections	Source	Altitude (m)
*V. serpens*	V1	Jammu, Jammu and Kashmir	2530
*V. serpens*	V2	Joginder Nagar, Himachal Pradesh	2286
*V. serpens*	V3	Kumaon, Uttarakhand	1789
*V. serpens*	V4	Kashmir, Jammu and Kashmir	2530
*V. serpens*	V5	Tehri Garhwal, Uttarakhand	1500
*V. serpens*	V6	Palampur, Himachal Pradesh	1472

**Table 5:** Sequence of SCAR primers, PCR condition, and product size.

SCAR Primers	No. of Base	Sequences (5ʹ–3ʹ)	G + C Content (%)	Annealing Temperature (°C)	Expected Product Size (bp)
Vio F	20	TAGCCTACAGTGCCCTTACG	55	58.89	285
Vio R	20	AATCGCACAGTCACCCAGTA	50	59.03	

## Supplementary Materials

The following are available online at https://www.mdpi.com/2223-7747/9/2/246/s1, Table S1: Effect of different plant growth regulators (PGRs) on leaf explants of *Viola serpens* for callus formation, Table S2: RAPD primers along with sequences, Table S3: Number of amplification products generated by 20 arbitrary primers in different accessions of *Viola serpens*, and Table S4: Commercial crude drug samples collected from the market place of New Delhi, India.
