# Supplementary material for: Development of an In Vitro Propagation Protocol and a Sequence Characterized Amplified Region (SCAR) Marker of Viola serpens Wall. ex Ging
Source: Plants (Basel). 2020 Feb 14;9(2):246. doi: 10.3390/plants9020246 (PMC7076368; doi:10.3390/plants9020246)
Supplement: Supplementary file 1 [file plants-09-00246-s001.pdf]

plants

MDPI

Supplementary Material

Table S1. Effect of different plant growth regulators (PGR's) on leaf explants of Viola serpens for callus formation.

Plant Growth Regulators	Concentrations (mg/L)	Callusing	No. of Days	Percentage of Explants Responded (%)
MS medium	-	-	50–60 days	0
MS + NAA	0.10	-		
	0.25	-		
	0.50	-		
	0.75	-		
	0.85	-	50–60 days	0
	1.00	-		
	1.50	-		
	2.0	-		
	2.5			
MS + BAP	0.10	-		
	0.25	-		
	0.50	-		
	0.75	-		
	0.85	-	50 days	
	1.00	-		
	1.50	-		
	2.0	+		15
	2.5	-		
MS + 2,4-D	0.10	-		
	0.25	-		
	0.35	-		
	0.40	+		
	0.50	-	25 days	
	1.0	-		10
	1.50	-		
	2.0	-		
	2.5			
MS + KIN	0.10	-		
	0.25	-		
	0.50	-		
	0.75	-		
	0.85	-	50–60 days	0
	1.00	-		
	1.50	-		
	2.0	-		
	2.5			
MS + BAP + 2,4-D	1.0 + 0.11	+		20
	1.5 + 0.12	-		-
	2.0 + 0.13	+	45 days	88.5
	2.5 + 0.14	+		75
	2.5 + 0.15	+		60
MS + BAP + IAA	1.0 + 0.25	-		-
	1.5 + 0.5	-		-
	2.0 + 0.5	-	25 days	-
	2.5 + 0.75	-		-
	2.5 + 1.0	+		12

MS + BAP + 2,4- D + KIN	1.5 + 1.0 + 0.2	+	50–60 days	40
	1.0 + 1.0 + 0.4	+		35
	1.25 + 1.0 + 0.5	-		-
	1.25 + 1.0 + 0.1	-		-
	1.5 + 1.0 + 0.3	+		45
MS + NAA + KIN	2.0 + 0.8	+	40 days	45
	2.2 + 0.8	+		38
	2.3 + 0.9	-		-
	2.0 + 0.5	-		-
	2.5 + 0.5	-		-

Table S2. RAPD Primers along with sequences.

SN	Sequences (5' → 3')	Primers
1	AGACGGCTCC	OPAA-01
2	GAGACCAGAC	OPAA-02
3	TTAGCGCCCC	OPAA-03
4	AGGACTGCTC	OPAA-04
5	GGCTTTAGCC	OPAA-05
6	TCAAGCTAAC	OPAA-06
7	CTACGCTCAC	OPAA-07
8	TCCGCAGTAG	OPAA-08
9	AGATGGGCAG	OPAA-09
10	TGGTCGGGTG	OPAA-10
11	ACCCGACCTG	OPAA-11
12	GGACCTCTTG	OPAA-12
13	GAGCGTCGCT	OPAA-13
14	AACGGGCCAA	OPAA-14
15	ACGGAAGCCC	OPAA-15
16	AAGCCTCGTC	BG-01
17	TGCGTGCTTG	BG-02
18	GACGGATCAG	BG-03
19	CACACTCCAG	BG-04
20	CACACTCCAG	BG-05

Table S3: Number of intensive amplification products generated by 20 arbitrary primers in different accessions of *Viola serpens.*

Primer code	Accession V1	Accession V2	Accession V3	Accession V4	Accession V5	Accession V6
**OPAA- 01**	5	5	6	5	5	7
**OPAA- 02**	---	---	---	---	---	---
**OPAA- 03**	9	8	6	9	8	9
**OPAA- 04**	8	9	9	8	8	6
**OPAA- 05**	---	---	---	---	---	---
**OPAA- 06**	---	---	---	---	---	---
**OPAA- 07**	4	5	6	5	5	5
**OPAA- 08**	5	6	5	4	6	5
**OPAA- 09**	6	8	6	8	8	6
**OPAA- 10**	4	6	5	4	3	4
**OPAA- 11**	---	---	---	---	---	---
**OPAA- 12**	---	---	---	---	---	---
**OPAA- 13**	---	---	---	---	---	---
**OPAA- 14**	---	---	---	---	---	---
**OPAA- 15**	---	---	---	---	---	---
**BG- 01**	---	---	---	---	---	---
**BG- 02**	5	6	6	5	6	6
**BG- 03**	---	---	---	---	---	---
**BG- 04**	---	---	---	---	---	---

**BG- 05**	---	---	---	---	---	---

Table S4: Commercial crude drug samples collected from the market place of New Delhi, India.

S. No.	Trade Name	Sample Code	Product Character
1	Banafsha	B1	crimpled leaves
2	Banafsha	B2	Powder
3	Banafsha	B3	dried stem
4	Banafsha	B4	powder
5	Banafsha	B5	crimpled leaves
6	Banafsha	B6	powder
7	Banafsha	B7	dried stem
8	Banafsha	B8	crimpled leaves
9	Banafsha	B9	powder
